# Procedural and educational interventions to reduce ventilator-associated pneumonia rate: two years of a national registration programme

**DOI:** 10.1186/2197-425X-3-S1-A429

**Published:** 2015-10-01

**Authors:** P Reper, D Dicker, W Aelvoet, P Damas, L Huyghens, P Haelterman

**Affiliations:** Brugmann University Hospital, Free University Brussels, Critical Care, Brussels, Belgium; Health Ministry, Brussels, Belgium; College of Physicians for Intensive Care Medicine, Health Ministry, Brussels, Belgium; University Hospital of LIège, Liège, Belgium; University Hospital Brussels (VUB), Critical Care, Brussels, Belgium

## Introduction

Belgian Public Health Organization is concerned with rates of hospital acquired infections like ventilator-associated pneumonia (VAP) or central line-associated blood stream infection (CLA-BSI). Implementing best practice guidelines for these nosocomial infections VAP and CLA-BSI has variable success in the literature.

## Objectives

This retrospective study was undertaken to see whether implementation of the evidence based practices as a bundle would influence compliance and reduce the rates of VAP without necessary resorting to more expensive interventions such as subglottic endotracheal (ET) tube suctioning or for example silver-impregnated ET tubes. We utilized easily collectable data to rapidly assess whether interventions already in place were effectively successfully applied. This avoided cumbersome data collection and review.

## Methods

Retrospective data review using National Healthcare Safety Network benchmarks. Compliance rates and VAP ratios were compared using z tests with P values < .05 considered statistically significant. This data review attempted to examine the impact of education campaigns, staff meetings, in-services, physician checklist, nurse checklist, charge nurse checklist implementation, systematic VAP bundle application and systematic protocols for oral care and sedation protocols. Additionally, VAP ratio could be registered by the participating centers but not always transmitted to the public ministry.

## Results

The general compliance for VAP bundle raised from February 2012 to December 2013

(P < 0.001). The incidence rate of VAP went from 21 occurrences/1000 vent days in February 2012 to 11.3 occurrences/1000 vent days in December 2013 (P < 0.001).

## Conclusions

Efforts to improve physician, patient, and staff education, and checklist implementation resulted in an increase compliance for VAP bundle, a local reflection on specific practices like oral care and sedation protocols and in a decrease in VAP ratio. This study confirms the applicability of best practice guidelines and suggests a benefit to the use of checklists proposed by Health Organization. We utilize a practical approach for examining the success of these changes.

